# Beyond the Cure: Unveiling the Silent Struggles of Breast Cancer Survivors in Hong Kong

**DOI:** 10.3390/healthcare14050647

**Published:** 2026-03-04

**Authors:** Alice Yip, Jeff Yip, Chun Sze Angela Chan, Zoe Tsui, Ka Man Rachel Yip, Yuen Han Mo

**Affiliations:** 1 S.K. Yee School of Health Sciences, Saint Francis University, 2 Chui Ling Lane, Tseung Kwan O, New Territories, Hong Kong, China; ztsui@sfu.edu.hk (Z.T.);; 2 Hong Kong Institute of Paramedicine, Hong Kong, China; jeffreyyip@gmail.com; 3 Department of Surgery, North District Hospital, Hong Kong, China; achan503@gmail.com; 4 Department of Social Work, Shue Yan University, Hong Kong, China; yhmo@hksyu.edu

**Keywords:** breast cancer, breast cancer survivors, emotional distress, resilience, traditional Chinese medicine, unmet care needs

## Abstract

**Background/Objectives:** Internationally, breast cancer incidence and survivorship are increasing. As the number of breast cancer survivors continues to rise, so does the demand for supportive care. This study aimed to explore how treatment experiences of breast cancer survivors in Hong Kong (HK) affect their unmet care needs, with a focus on how Chinese culture influences their journey. **Methods:** This phenomenological qualitative study engaged a purposive sample of 28 breast cancer survivors in HK through semi-structured interviews. Data were analyzed using Colaizzi’s seven-step method to interpret their lived experiences. **Results:** Four key themes emerged: (i) carrying the burden in silence: the isolation of self-preservation; (ii) beyond the clinic: making medical advice fit into everyday routine; (iii) bridging two worlds: resilience through the integration of Traditional Chinese Medicine and Western Care; and (iv) reclaiming femininity: women helping women heal. **Conclusions:** This study provides an understanding of breast cancer survivors’ experiences and offers insights into delivering more realistic services. Basically, it extends survivorship knowledge by demonstrating how integrating cultural values into clinical care bridges the gap between medical treatment and holistic well-being.

## 1. Introduction

Globally, breast cancer affects women more than any other cancer, with nearly 2.3 million diagnoses in 2022 [1]. Breast cancer significantly impacted Hong Kong (HK) women in 2022, accounting for 28.6% of all new cancer cases and ranking as the most common cancer diagnosis among females [2]. As more women survive breast cancer, and with increasing incidence rates, a larger population of females is living with the disease’s long-term effects. Enhanced early detection and treatment modalities have contributed to improving breast cancer survival outcomes, consequently necessitating increased provision of supportive care involvement, resources, and services for this expanding population [3].

Finishing treatment does not mean the struggle is over; breast cancer survivors often cope with serious physical and emotional issues that go unnoticed. Despite the relief of finishing treatment, many women find their return to normal life blocked by persistent pain, exhaustion, and other physical challenges [4,5,6,7]. At the same time, survivors face a heavy emotional toll, struggling with body image issues, the constant fear of the cancer returning, loneliness, and the stress of overcoming medical bills [8,9,10,11]. Because these physical and emotional struggles can often lead to depression, anxiety, and post-traumatic stress disorder, it is vital that breast cancer survivors have long-term support through counseling, support groups, and honest talks with healthcare providers [12].

For women facing breast cancer, distress is a complicated mix of physical, emotional, and spiritual challenges that can range from normal concern to serious mental health issues, all of which affect how well they deal with their diagnosis and treatment [13,14]. Breast cancer survivors often find their struggles are made worse because they are not getting enough support from their healthcare providers [15,16,17]. Psychological distress is not uncommon among survivors, often persisting long after treatment ends, such as fatigue, anxiety, panic, and depression [18].

Unmet care needs reveal a disparity between the healthcare services provided and the expectations of breast cancer survivors [19]. This emotional distress can be attributed to the burden of physical symptoms and the ongoing challenge of navigating life with unmet supportive care needs, including psychosocial support and information about managing long-term side effects [20,21]. About 25% of breast cancer survivors suffered from serious issues like mood disorders and isolation because their emotional needs were not being met [15,22]. Compared to women who have not had cancer, breast cancer survivors were much more likely to struggle with mental health challenges like depression, anxiety, and suicidal thoughts [23,24]. Although breast cancer survival rates have improved, the post-treatment phase remains difficult for many women. To address their unmet needs, we must prioritize research into the challenges of this transition [25,26]. Just like in Western countries, studies showed that breast cancer survivors in Korea often struggle with ongoing sadness, low energy, and a loss of interest in their daily lives. These symptoms added up to significant emotional distress, showing that regardless of cultural background, the mental burden of surviving cancer is a struggle that deeply affects overall quality of life [20,23].

Emotional well-being is paramount for breast cancer survivors, as remaining distress can slow rehabilitation and escalate dependency on external support [17]. To reduce psychological risks like depression or anxiety, comprehensive care must target unmet needs across physical, financial, spiritual, and psychological realms [4,11]. Since survivorship is not an unchanging experience, interventions must be tailored rather than uniform [27]. While a vigorous body of qualitative inquiry has reported the way of breast cancer survivorship in Western circumstances, it emphasizes personal autonomy and post-traumatic growth; these systems often fail to fully express the refined reality of Chinese survivors, for whom identity reconstruction is totally bound to collective family obligations and culturally specific fortitude and resilience. Understanding these cultural nuances is especially important in places like HK, where family ties and traditional values play such a huge role in how people handle their health [28]. In these communities, cancer is not just one person’s battle because the whole family shares the emotional weight and the financial cost. Consequently, maintaining familial stability is integral to the recovery process, requiring comprehensive support interventions that encompass not only the patient but also the multifaceted needs of the whole family [29]. While current knowledge has effectively classified the prevalence of unmet supportive care needs and emotional distress among Chinese survivors, it has been less successful in explaining the elementary mechanisms that sustain these deficits. Specifically, it remains unclear how the cultural dominance to ‘preserve face’ and carry on social roles specifically inhibits a woman’s ability to arrange a new post-cancer identity, thus leaving specific psychosocial needs persistently unmet. Contemporary studies increasingly indicate survivorship not as a return to normalcy, but as a prolonged period marked by persevering distress and the difficult task of meaning reconstruction [7,11,14,15,19,20,22]. By investigating these dynamics within a Chinese situation, this study focuses on situating its findings beyond an entirely localized phenomenon, demonstrating instead how specific cultural frameworks modify the universal, long-term challenges of life after breast cancer, with a focus on how Chinese culture influences their journey.

### Theoretical Framework

The post-treatment trajectory for breast cancer survivors is frequently characterized by a group of unmet supportive care needs that threaten to compromise long-term well-being. To show how survivors reduce these difficulties, this study uses the Resilience in Illness Model (RIM) as its main guide [30]. RIM provides robust information for examining how protective factors, specifically fearless coping, family hardiness, and social integration, interact to buffer the deleterious effects of illness-related distress [31]. Instead of seeing survivorship as a fixed point of recovery, this framework views it as an ongoing journey of bouncing back and fitting the pieces of life back together. By operationalizing resilience as a modifiable capacity rather than an inherent trait, this study investigates how survivors leverage adaptive behaviors to transcend physical restraints and spontaneous generation determination to maintain optimal health throughout the lifespan. This study aimed to explore how treatment experiences of breast cancer survivors in HK affect their unmet care needs.

## 2. Materials and Methods

### 2.1. Design

Grounded in a descriptive phenomenological approach, this study seeks to bracket prior theoretical assumptions to respectfully delineate the sensitive, lived experience of survivors’ emotional distress and unmet needs entirely as they recognize them. Semi-structured interviews were conducted to facilitate a flexible, in-depth exploration of these phenomena within participants’ specific contextual realities [32].

### 2.2. Sample

This study purposefully recruited 28 women aged 18 and older in HK who completed primary breast cancer treatment (diagnosis, surgery, and adjuvant therapies) between 2024 and 2025. Recruitment kept going until data saturation. The majority of participants were ethnic Chinese individuals utilizing the public healthcare system, consistent with the demographics of the local population. Women who had finished treatment within one year were found for the study by referring breast cancer nursing specialists and local support groups like “Ho Chi Club’ and ‘Yin Hong Club’ for recommendations.

### 2.3. Ethical Consideration

Approved by a local medical institute’s Research and Ethics Committee, this study adhered to all relevant guidelines (HRE23004). Prior to the interviews (in-person or phone), participants were fully informed of the study’s goals and provided with written consent. Participants were volunteers and allowed cancel anytime. Recognizing the emotional vulnerability inherent in discussing cancer recovery, researchers prioritized participant safety by creating a distress arrangement that permitted immediate pausing of interviews during moments of difficulty and ensured a clear referral pathway to professional counseling services for any participant who needed further support. Data confidentiality was secured through anonymization. Additionally, the study confirmed each participant’s comfort with communicating in Chinese.

### 2.4. Data Collection

Between February and October 2025, a single specialist (Y.A.) in breast cancer research handled all the Chinese-language interviews to maintain consistency. To respect everyone’s preferences, the one-on-one sessions were held either face-to-face or over the phone, depending on what worked best for the participant. Each session lasted between 50 and 120 min and was audio-recorded and transcribed verbatim after obtaining written consent. Every participant was interviewed using a standard guide (Supplementary Table S1) approved by experts in qualitative research and psychology. The complete interview guide used in this study is open for review. Throughout the process, field notes were kept to record the small details of the environment, which added valuable depth to the final analysis. Throughout the study, the researcher prioritized participant safety, actively monitoring for signs of emotional distress and remaining prepared to offer immediate support.

### 2.5. Data Analysis

The semi-structured interviews were originally conducted in Chinese to allow participants to demonstrate themselves in their native language. These sessions were recorded and transcribed verbatim. To ensure the unity of the data during the transition to English, a correct translation protocol is required. While the initial analysis was performed on the original Chinese transcripts to maintain cultural nuance, selected excerpts considered for publication were translated into English. To reduce interpretative bias and establish cross-cultural validity, it is advisable that these translations undergo a back-translation process or review by an independent bilingual expert. This step serves as a quality control measure to prove that the English quotes accurately demonstrate the participants’ original desire without the researchers’ subjective influence.

Two researchers (A.Y. and J.Y.) transcribed individual interview recordings verbatim using NVivo software (NVivo Version 12, QSR International, Burlington, MA, USA). The data, including field notes, were analyzed using Colaizzi’s seven-step phenomenological method [33]. First, the team got to recognize the dataset by reading it over and over. This process helped uncover key themes naturally, without any need to decide on categories beforehand. After setting aside their own biases, the researchers interpreted the data units (bracketing personal biases) and organized them into starting codes. By discussing it over again and again, the researchers improved the draft codebook. It ended up being a mix of planned categories and new ideas that popped up during the reading. These codes were combined to fully explain the participants’ experiences, which eventually brought the main themes and sub-themes to light. To guarantee accuracy, participants checked their own transcripts to confirm their words were right, a step called member checking. Finally, the researchers compared the themes with the initial data to confirm that their analysis was correct, ensuring the final structure accurately represented a mix of all the research team’s coding approaches. In concurrence with Colaizzi’s method, the research team maintained a sustained practice of reflexivity throughout the analysis to critically examine the researchers’ own expectations, verifying that the interpretation of these sensitive, culturally layered narratives remained faithful to the participants live phenomena.

Data saturation was set on functionally through an iterative process of concurrent data analysis. A stable thematic map comprising all final themes and sub-themes was confirmed by the 26th interview. To show this saturation, two additional interviews were conducted. Analysis of participants 27 and 28 confirmed that no new meaning units or thematic differences appeared, serving as a verifying step that the dataset had realized sufficient information power.

### 2.6. Rigor

Data analysis employed Colaizzi’s phenomenological method [33]. Three investigators (A.Y., J.Y., and Z.T.) independently evaluated transcripts to extract significant statements and derive themes (Supplementary Table S2). The study’s reliability was confirmed by following Lincoln and Guba’s guidelines, which included member checking findings with participants and gaining a team consensus [34]. Follow-up discussions made it clear how the sub-themes were alike and how participants were unique. Furthermore, adherence to the Consolidated Criteria for Reporting Qualitative Research (COREQ) checklist ensured reporting transparency and quality [35].

## 3. Results

Twenty-eight Chinese breast cancer survivors receiving post-operative follow-up in HK public hospitals were recruited (Table 1).

### 3.1. Theme 1: Carrying the Burden in Silence: The Isolation of Self-Preservation

Even after finishing tough treatments like surgery, hormone therapy, and chemotherapy, nineteen participants feel a deep gap in their recovery. While their bodies may be healed, their minds often remain unsettled by a constant fear that the cancer will return. This unmet need for emotional support leaves participants constantly on guard, mistaking normal aches for signs of danger. Without proper guidance to handle this anxiety, ‘beating’ cancer did not bring peace, leaving many to face uncertainty and fear alone.

*“They tell me I’m cured, but my chest feels like a house that has been burglarized; the scars mark exactly where they broke in to save me. I live with constant health anxiety; every minor ache feels like a serious threat, and every mammogram brings terror. I may be physically whole, but mentally, I am still holding my breath. I am learning to love my changed reflection, but it is hard to dance when you are constantly waiting for the music to stop.”*

(P07)

Although this participant accepted her physical scars with her husband’s support, three participants fiercely maintain their health and independence to avoid becoming a burden on their family again.

*“I declined reconstruction. My husband loves me as I am, scars and all. Yet, silence brings the cold fear of recurrence. Every cough feels like a threat. My motivation is not self-centredness; I remember their exhaustion during my chemotherapy, my husband’s cancelled meetings, my son’s tired eyes. I never want to be that burden again. So, I exercise religiously and guard my health fiercely. I stay strong not for myself, but to ensure I never have to be carried by them again.”*

(P01)

Although the participant viewed her changed reflection as a testament to survival and endured the suffocating humidity and the pain of her prosthesis in silence to preserve her dignity, her stoic guarding of her health solely to spare her family revealed a profound, unspoken longing for support and a deep-seated fear of becoming a burden that remains unaddressed.

*“At my age, the mirror reflects survival, not vanity; I have made peace with the altered shape of my chest, viewing it merely as the vessel for my beating heart. I fight through the daily struggle of Hong Kong’s humidity, making my prosthesis feel sweaty and dirty because staying independent is worth it to me. I just want to stay healthy so I do not end up becoming a heavy weight on my family’s shoulders. I hold my silence like a shield, guarding my private battle so I can stand tall and alive without needing anyone to feel sorry for me.”*

(P12)

### 3.2. Theme 2: Beyond the Clinic: Making Medical Advice Fit into Everyday Routine

Even though participants were grateful for their medical treatments, they still felt a big lack of practical help in their daily lives. Unmet care simply means not obtaining the specific, practical advice needed to carry on daily life at home. Participants wish for more than just medical facts; they need tailored life hacks from nurses to bridge the gap between clinical treatment and comfortable living.

*“I can’t fault the doctors for saving my life; the surgery and the chemo went exactly as planned. But when I walked out those hospital doors, I felt incredibly alone. I did not need another pamphlet on survival rates; I needed someone to tell me how to sleep when my chest felt tight or what specific balm would stop my lips from cracking until they bled. I was drowning in medical facts, but I was starving for those simple, practical tips that would help me get through a Tuesday afternoon without breaking down.”*

(P21)

Though participants valued their nurses’ initial care, they felt follow-up appointments were too rushed for meaningful conversations. They wished for more time to receive the deep, personal guidance they needed.

*“My nurses were wonderful, they provided so much information, especially regarding hormone therapy, and truly took great care of me. However, the follow-up appointments always feel rushed. Just as I start to feel comfort enough to ask the real questions about living in this new body, our time is up. I find myself wishing our sessions could last longer. It would be a dream to just sit with them without watching the clock, so I could really explain what I’m going through and get the deep, personal guidance I desire.”*

(P04)

Participants often felt physically unsettled and abandoned after treatment, lacking professional advice on how to safely incorporate Traditional Chinese Medicine (TCM) into their recovery. Due to this uncertainty, they strongly desire their trusted nurses to step in and provide the necessary guidance to bridge the gap between conventional care and holistic healing.

*“While I am grateful the tumor is gone and for the scheduled follow-ups, I still feel lost in the messy aftermath of treatment. I wish I had more time with my nurses because I trust them completely. I am interested in trying Traditional Chinese Medicine to help my recovery, but I need guidance on safety and relevance. Because I have such confidence in my nurses, getting their specific advice on this new path would give me the courage to move forward.”*

(P23)

### 3.3. Theme 3: Bridging Two Worlds: Resilience Through the Integration of Traditional Chinese Medicine and Western Care

For most participants, resilience is found in harmonizing modern oncology with cultural wisdom. While they rely on Western treatments for survival, they often feel an unmet need for holistic symptom management. To deal with side effects like nausea, tiredness, and lymphedema, these participants looked to TCM for help. They thought that methods like massage, ear acupuncture, and food therapy were essential for restoring balance, rather than just treating symptoms. By using these ancient traditions, they were taking charge of their energy and regaining a recovery that recognized both their bodies and their culture.

One participant enjoyed the traditional dishes that her daughter made for her; she regained her strength and showed just how healing cultural food can be. This participant delineated the difference between just surviving and truly being well, explaining that Western medicine treated her cancer but left her body feeling completely drained.

*“Chemotherapy saved my life but left me weak. While I trust the doctors, true healing came from my daughter’s traditional soups. The medicine treated cancer, but the familiar warmth of red dates and ginger restored my spirit. To feel whole again, I needed both medical science and the nourishment of my culture.”*

(P19)

Dealing with severe side effects that the doctor overlooked, twelve participants sought relief and regained their physical strength through TCM. They utilized massage and acupuncture because they deeply believed that fixing the body’s energy flow would heal the real problem rather than just hiding the pain. One of the participants said the following:

*“The lymphedema left my arm swollen and heavy, and the numbness in my fingers kept me awake at night. Although my doctors insisted everything was normal, I knew something was wrong and refused to simply accept the pain. I turned to massage and acupuncture, and slowly, I felt the energy begin to flow again. It feels like I am finally addressing the root of the problem rather than just masking it, and that gives me hope.”*

(P07)

Struggling with post-surgical emotional distress and insomnia, five participants rejected further pharmaceutical dependence in favor of the gentle, non-invasive practice of ear acupuncture. The application of acupressure seeds provided them with a vital sense of autonomy and spiritual grounding, allowing them to address their mental health needs through a culturally familiar and comforting method. A participant stated the following:

*“After the surgery, my spirit felt scattered, and insomnia became my constant companion. I was too afraid to take more sleeping pills, so I tried ear acupuncture instead. Pressing those tiny seeds on my ear gave me a sense of control I had not felt in months. It’s a gentle way for me to find peace and rest that just feels natural for my body. I think seeing a Chinese medicine practitioner is too expensive, I can’t afford it.”*

(P27)

### 3.4. Theme 4: Reclaiming Femininity: Women Helping Women Heal

For the Chinese participants, cultural habits often made it hard to share their struggles openly, which unfortunately left them suffering in loneliness and unsupported. In peer support groups, participants found a shelter to safely express themselves, shared coping strategies, and remember their feminine identity through cultural traditions like making cosmetics or sewing Cheongsams (Qipao). However, because budget cuts are threatening these vigorous programs, participants expressed that they urgently need more funding from the government and non-profits to keep providing this vital sense of community and self-worth.

Furthermore, participants did not desire to stress out their families with their own emotional struggles, mostly because their culture expects them to stay strong and keep quiet at home. Because the peer support group offered such a unique and safe place for emotional support and self-care, they highlighted that funding was absolutely necessary to keep it going. A participant expressed the following:

*“At home, I hide my pain and my fear of recurrence because I don’t want to burden my family. But our Wednesday group is different. I remember when the nurse taught us to make organic lipstick to avoid the chemicals in store brands, I just started weeping. It was not really about lipstick. It was the feeling that someone finally cared about both my beauty and my safety. We desperately need more resources for these sessions. Being with these ‘sisters’ is the only time I feel safe enough to speak from the heart.”*

(P04)

One participant stated how hard she struggled with her body image and feeling like a woman after her mastectomy, even throwing away clothes that showed off her figure. She explained how specialized activities in tailoring Qipao helped restore her dignity, arguing that financial support is essential to continuing these restorative programs.

*“After the surgery, I felt like I was not a real woman anymore. I threw away all my tight clothes because I was ashamed of my chest. Our support group brought in a tailor who showed us how to adjust Qipaos so we could feel elegant and proud instead of broken. But now they say we might not have funds for the next activities. Please, do not take this away. That activity did not just give me a dress; it gave me back my dignity.”*

(P17)

A participant distinguished between medical treatment and the emotional isolation she experiences, noting that the cultural norms of silent endurance often invalidate her side effects. She asserted that the peer group validated her physical symptoms and provided practical coping strategies, urging external organizations to sponsor these meetings to prevent the loss of this communal support system.

*“My doctor treats the tumor, not the loneliness. While culture demands silent endurance, my peer group validates my pain and proves it is not just in my head. We share healing recipes and support, but we lack the budget for simple wellness activities. These gatherings are true lifesavers. If the government understood how essential this community connection is for our recovery, they would surely offer the funding we need to keep going.”*

(P08)

## 4. Discussion

This study investigated the real-life experiences of breast cancer survivors in HK, seeking to understand how the challenges of the disease interact with traditional Chinese cultural beliefs regarding illness, femininity, and family to influence their care needs. Supported by the resilience in RIM, this study examines how Chinese breast cancer survivors in HK navigate recovery by intertwining cultural resilience with Western treatment [30,31,36]. Beyond physical trauma, survivors utilized Confucian values as active mechanisms of resilience. Social integration was strengthened by prioritizing family harmony, where protecting loved ones from worry provided survivors with purpose. Similarly, traditional gender roles, while physically demanding, offer a stabilizing identity beyond that of a “patient” [37,38]. Furthermore, courageous coping was demonstrated through TCM beliefs, allowing women to address imbalances in *qi* and reclaim personal agency often diminished by clinical protocols. Ultimately, these cultural touchstones bridge the gap between medical treatment and holistic well-being, fostering a healthy, culturally grounded recovery. It is significant to explore this now because ignoring these specific cultural preferences could lead to serious problems. Requiring a comprehensive understanding of the mechanisms by which participants of this study transition from vulnerability to resilience, healthcare systems are liable to deliver insufficient support. Addressing this is necessary to establish that there is enough funding for peer support activities and to provide trustworthy, evidence-based guidance on TCM. If these cultural and familial requirements are ignored, current support structures may inadvertently undo the very protective mechanisms, such as trust in TCM and family cohesion, that participants rely upon to restore health. Therefore, this research advocates for a more holistic integration of Western and TCM paradigms to facilitate resilience in HK. The RIM perspective discloses how people draw strength from both scientific and their culture to aid their recovery [30,31,36]. This discussion distinguishes the findings of this study, showing how healthcare providers and policymakers can connect standard medical rules with the real-life recovery experiences of Chinese patients.

This study reveals that participants live with overcoming fear of their cancer recurrence, causing them constantly to worry that normal body sensations are signs of the breast cancer returning. This anxiety is made much more unacceptable by Chinese cultural expectations, which pressure patients to keep their emotions to themselves in order to preserve peace within the family [39,40,41]. To avoid being a burden on their loved ones, the participants select to stay silent about their distress regarding hormone therapy side effects and the possibility of a setback. Even though this lack of reaction fits their culture, it leaves them feeling extremely lonely because they cannot be open about their true feelings at home [39,40,41]. Hence, the research indicates a critical need for a psychological space where people feel culturally safe enough to express their emotions [42]. The gap between how tough participants look on the outside and how much they are struggling on the inside suggests that care plans are not good enough if they only fix the body but disregard the heavy mental burden of suffering in silence. Therefore, clinical interventions must account for this cultural phenomenon, providing outlets for fear of cancer recurrence that do not compromise participants’ perceived obligations to protect their family from emotional distress [40,42].

While participants of this study acknowledged the curative efficacy of standard oncological interventions, including chemotherapy, hormone therapy, radiotherapy, and surgery, their narratives disclose a critical opposition between clinical success and the lived experience of survivorship. A remarkable gap persists in the provision of pragmatic, longitudinal support, characterized here as ‘unmet care needs’ [19,43]. Specifically, this is defined not as a lack of medical intervention, but as the absence of personalized, actionable strategies for managing the domestic reality of recovery [43,44]. Participants expressed a distinct preference for guidance that transcends generic medical education, seeking instead tailored lifestyle adaptations, or ‘practical wisdom’ from nursing professionals to bridge the chasm between clinical protocols and the restoration of daily comfort [45,46].

A primary driver of this unmet need is the debilitating constellation of post-treatment sequelae, including insomnia, persistent pain, cancer-related fatigue, and chemotherapy-induced peripheral neuropathy (numbness of hands and feet) [47]. These symptoms often lead people to try TCM upon their regular treatment, a choice driven by their cultural belief in healing the whole body to restore balance [37,48]. However, it’s often difficult to predict exactly how TCM will interact with modern medical treatments. Participants felt physically weak and left on their own after finishing their main treatment, with no clear guidance from healthcare professionals on how to safely combine both medical systems. Without professional oversight, patients are at risk for dangerous side effects or bad reactions between herbs and drugs, disclosing a major safety gap in how we currently care for survivors.

Even though participants recognized their initial nursing care, short follow-up appointments seldom gave them enough time to properly discuss their complex needs for integrative medicine [49,50]. Since these appointments were so rushed, survivors could not get the deep, personal guidance they needed to really cope with their symptoms [51]. These results underline just how important nurses are as trusted cultural guides. Participants clearly expressed a strong hope for nurses to go beyond just medical duties and offer evidence-based advice on holistic healing.

Theme 3 delineates that participants actively take charge of their recovery by combining Western cancer treatment with TCM to better cope with side effects like pain, insomnia, fatigue, and peripheral neuropathy that standard care does not fully fix. For these survivors, healing means restoring balance and energy rather than just preserving the disease, but they struggle to find evidence-based guidance on how to safely combine treatments without causing harmful side effects [37,52]. Even though participants turned to gentle methods like auriculotherapy and food therapy to regain control over their bodies and rely less on medication, there is still a major gap in care where they hopelessly need trusted nurses to guide them through these cultural treatments safely [53,54]. Ultimately, formalizing these integrative oncology models would not only respect the patient’s cultural wisdom but also ensure that the synthesis of Western curative efficacy and holistic TCM philosophy is managed safely, thereby enhancing long-term quality of life through professionally supported, culturally congruent self-care [37]. Having survived cancer, participants of this study are fueled by a powerful will to live. They are dedicated to strengthening their bodies and minds, actively seeking out every possible strategy to ward off recurrence. These survivors manifest unbelievable strength by dedicating the rest of their lives to valuing and guarding the future they fought so hard to save.

The high use of TCM among participants should not be interpreted entirely as a decline of Western biomedicine, but rather as an attempt to regain bodily autonomy in a disintegrated healthcare system. In the setting of HK, where acute oncology care is vigorous but transitional survivorship care is often fragmented, TCM practitioners fill a critical space by giving holistic, healthy attention that is lacking in standard follow-ups. This corresponds with the Self-Regulation Model of Illness, where survivors actively look for restoration equilibrium [55]. The unmet need here is fundamentally a structural gap: the biomedical system manages breast cancer, while survivors are forced to look elsewhere, often to TCM, to handle the person (vitality and balance). This underlines the crucial need for an integrative care model within HK’s public health framework.

The integration of TCM and Western care by HK survivors represents a narrative form of cultural resilience, where patients reasonably adapt treatments to retrieve agency. Yet this resilience often hides a continuing emotional burden. Aligning with recent longitudinal evidence [56,57], our findings validate that distress develops rather than settling post-treatment. This cultural tendency toward emotional suppression mirrors vulnerabilities in a systematic review [58]. Clinical practice should look beyond physical recovery to convey these deep-seated, often silent, supportive care needs.

The study participants explained that peer support groups are essential because they often feel emotionally isolated by cultural pressures to stay strong and keep family problems private [59,60]. Since they feel like they cannot exhibit their pain at home without upsetting their families, these peer support groups become essential safe spaces where they can finally talk openly about their feelings and the side effects of their treatment [61]. Moreover, cultural activities like making traditional Cheongsam dresses are incredibly important for helping women feel feminine and dignified again after mastectomy, giving them a way to see themselves as more than just cancer patients [62]. However, a lack of funding places these essential programs at risk of disappearing. To fix this, participants urgently request steady funding from the government and non-profits that treat these activities as needed mental health services, not just fun extras. We offer cost-cutting measures with online support tools, teaching families that needing help is not a burden, and ensuring that patients are connected with peer support groups before they leave the hospital. Protecting these resources is essential to help survivors maintain their dignity and emotional resilience, as getting better while trying to balance different cultural expectations can be really tricky [63].

### 4.1. Limitations

These findings might not apply to all Chinese people since the study used a small number of female participants in just one setting. The findings may be subject to selection bias, supporting healthier survivors with greater socioeconomic resources, as well as recall influence regarding past events. Additionally, cultural pressure to be polite may stop participants from criticizing doctors, hiding their true needs.

### 4.2. Implications for Practice

The findings of this study emphasize a critical need to realign clinical practice and nursing education with the evolving healthcare landscape in HK. Although the new TCM Hospital in Tseung Kwan O is a major upgrade for the healthcare system in HK, it clearly indicates that the workforce is not quite prepared to keep up with such advanced facilities.

To utilize these findings, healthcare providers in HK must embrace a culturally responsive survivorship model that traverses biomedical care with TCM. Oncology and breast care nursing specialties should acquire training to carefully discuss TCM usage, framing it as a verified coping mechanism to enable open dialogue about emotional well-being. Survivorship programs need to perform routine screening for ‘silent’ distress, recognizing that cultural norms often conceal anxiety, while organizing peer support networks that verify these specific lived experiences. At a policy level, formally integrating TCM consultations into standard cancer follow-up care would validate patients’ holistic self-management strategies, ensuring that psychological support is as attainable and normalized as physical treatment.

Currently, local nursing curricula predominantly relegate TCM to theoretical coursework, resulting in a workforce that possesses abstract knowledge but lacks the practical competency to navigate the dual-system care model effectively. To ensure patient safety and culturally congruent care, a systematic reform is required to bridge the gap between biomedical protocols and the reality of TCM usage among survivors.

To anchor these missing pieces, we suggest taking the following steps:

- **Development of TCM Nursing Standards:**
  - *Defining Competency:* Since HK currently lacks a standard for TCM nursing, regulatory groups need to work together to clearly define what nurses in integrative settings are allowed to do and what skills they need to have.
  - *Standardization:* We require creating clear guidelines so that nurses know exactly how to record, evaluate, and give advice on TCM treatments, making sure everyone in the region gets the same high-quality care.
- **Establishment of Evidence-Based Learning Programs:**
  - *Content Focus:* We require the building of new programs that use safe, holistic methods to help patients handle persistent side effects like sleep problems, tiredness, and nerve pain.
  - *Interdisciplinary Curation:* Interdisciplinary collaboration includes healthcare professionals, especially nurses and TCM experts, who should work together to generate guides that help patients safely use natural therapies without interfering with their medications.
- **Transforming of Nursing Curricula:**
  - *Shift from Theory to Practice:* Instead of treating TCM as just a minor or optional topic, nursing programs should require updating their classes to make it a central, practical skill that every nurse learns.
  - *Addressing the “Theory–Practice-Gap”:* Training institutions must realize that simply knowing the facts is not enough to make the complex clinical decisions needed in modern, integrative healthcare.
- **Application of “Reality-Based” Clinical Placement:**
  - *Mandatory Rotations:* HK training programs urgently require the inclusion of hands-on internships in TCM units or integrative clinics, something most of them currently lack.
  - *Skill Acquisition:* Training needs to focus on real-world skills, granting nurses the opportunity to practice holistic assessments and basic TCM treatments under supervision.

## 5. Conclusions

This study explains the difficult path breast cancer survivors in HK face, indicating how their culture and treatment history directly affect the support they still need but are not getting. The results illustrate that survivors deal with their constant fear of cancer recurrence by combining TCM with Western treatments to help fix side effects and feel balanced again. As their culture discourages burdening family members, many people cache their emotional pain and body image worries, which makes peer support groups a necessary place for them to speak freely. Survivors actively seek out their own paths to resilience, finding ways to regain their strength and well-being in order to alleviate the impact of treatment side effects. Although survivors appreciate the kind care and advice they receive from medical professionals, they clearly desire TCM options that are officially backed by their medical teams. Therefore, this study recommends managing a care approach that respects cultural differences by combining emotional support with advice on TCM.

To turn these insights into action, future research must look beyond the immediate post-treatment period. We specifically recommend longitudinal studies to follow how emotional distress and reliance on TCM expand over time, adjoining intervention-based research to test culturally tailored support programs. Generating this evidence is the necessary next step toward designing clinical guidelines that respectfully integrate traditional practices, eventually ensuring a more complete and holistic recovery for these survivors.

## Figures and Tables

**Table 1:** Characteristics of participants.

Characteristics		Mean (Min–Max) [Median/Standard Deviation (σ)]	n	(%)
*Age*		57.8 (from 45 to 75) [59/σ = 7.3599]		
	45–49		4	14
	50–54		6	21
	55–59		5	18
	60–64		8	29
	65–69		4	14
	70–74		0	0
	>75		1	4
*Marital status*				
	Single		7	(25)
	Married		14	(50)
	Divorced/separated		7	(25)
*Education level*				
	Primary		0	(0)
	Secondary		16	(57)
	Bachelor’s degree or above		12	(43)
*Household*				
	Single-person household		6	(21)
	Family household		22	(79)
*Employment*				
	Full-time		11	(39)
	Part-time		8	(29)
	Retired/unemployed		9	(32)
*Reason for Surgery*				
	Beginning treatment for invasive breast cancer		20	(71)
	Diagnosed with a localized breast carcinoma		7	(25)
	To minimize the threat of developing breast cancer		1	(4)
	Unclear		0	(0)
*Hormone Treatment*				
	Yes		24	(86)
	No		4	(14)
*Religion*				
	Buddhist		5	(18)
	Christian		6	(21)
	Catholic		3	(11)
	Nil		14	(50)

## Data Availability

The data that support the findings of this study are available from the corresponding author upon reasonable request.

## Supplementary Materials

The following supporting information can be downloaded at: https://www.mdpi.com/article/10.3390/healthcare14050647/s1.

## Abbreviations

The following abbreviations are used in this manuscript:

COREQ	Consolidated Criteria for Reporting Qualitative Research
HK	Hong Kong
RIM	Resilience in Illness Model
TCM	Traditional Chinese Medicine
