# Supplementary material for: Beyond the Cure: Unveiling the Silent Struggles of Breast Cancer Survivors in Hong Kong
Source: Healthcare (Basel). 2026 Mar 4;14(5):647. doi: 10.3390/healthcare14050647 (PMC12984250; doi:10.3390/healthcare14050647)
Supplement: Supplementary file 1 [file healthcare-14-00647-s001.zip › healthcare-4109909-supplementary.pdf]

Supplementary Table S1. Interview Guide Questions.

1.	Can you tell me how you felt once the treatment was over
2.	What do you think causes these feelings
3.	What kind of support did the medical team give you once you were done with treatment
4.	Are you still searching for a solution to that
5.	What do you do to make yourself feel better when you are feeling stressed
6.	Do you wish you had more advice on this
7.	What health problems are you dealing with right now that you need help with
8.	Would you like support in feeling closer to the people around you
9.	Do you feel like you need advice on how to handle things with your family or close friends
10.	Do you find yourself needing support to get through your daily routine
11.	Does your memory feel the same as it did before you got sick

Supplementary Table S2. Approaches to Establishing Trustworthiness in the Study.

**Standards for Rigor**	**Specification**	**Approaches to reinforce the reliability of the findings**	**Practical recommendations**
Credibility	To strengthen the validity of the analysis	To look deeply at each participant's experience over time and in different ways	- To ensure the credibility of our findings, we employed the strategy of prolonged engagement. By meeting with the participants multiple times, we established a foundation of trust that encouraged them to go beyond socially desirable responses, a common hurdle in Hong Kong's health-focused environment. This approach allowed us to capture the reality of their dietary habits and lifestyles, including the imperfect moments, resulting in a dataset that is both true and robust<br>- By mixing different types of data, specifically interviews and personal photo-diaries, we were able to look behind the curtain of participants' lives. This strategy helped us spot the difference between their stated intentions and their actual behaviors. For instance, we discovered a gap: while many participants expressed a desire to stick solely to Western medicine, their diaries revealed they were frequently turning to Traditional Chinese Medicine (TCM) after major procedures like mastectomy, chemotherapy. Unearthing these hidden tensions ensures our findings are credible and reflect the complex reality of healthcare in Hong Kong
		To carry out detailed, open-ended inquiry sessions	- To ensure the quality and validity of our data collection, the interview guide emphasized a systematic refinement process immediately following ethical approval. The questions were first subjected to peer review during two separate research team meetings, where we focused on improving clarity and ensuring strict alignment with our research goals<br>- Two pilot interviews followed to assess flow and question quality. As these tests proved the guide was effective without needing revision, the pilot data were retained for the main analysis
		To establish the credibility of the findings by prioritizing the participants' viewpoints	- Our team was comprised of researchers who brought a combination of advanced academic training and hands-on experience with qualitative methodologies<br>- Senior research over six years of experience oversaw the project, mentoring the team and supervising their roles to ensure our data collection remained consistent and high-quality
		To ensure trustworthiness through the systematic preservation of all raw	- Throughout the interviews, we took field notes to record important details like body language and the setting. We analyzed these notes at the

		data for future reference	same time as the interview transcripts, which helped us better understand the participants' narratives and ensured our results were more reliable
		To maintain interpretive validity through regular peer debriefing sessions designed to challenge and refine emerging themes	- - To ensure analytical rigor, the research team held weekly debriefing sessions with expert Fellows from the Hong Kong Academy of Nursing and Midwifery. These meetings offered a critical external viewpoint, allowing us to challenge our emerging interpretations and uncover any unexamined statements
			- -
Dependability	To verify the reliability of the findings	To offer a comprehensive and contextualized account of the methodological procedures	- - The selected studies provided enough methodological detail to create a clear audit trail, thereby strengthening the dependability of their results
		To ensure the study's dependability by keeping aa rigorous log of the research trajectory	- The entire team kept a detailed audit trail of all data collection activities, which helped us safeguard the dependability of our research process<br>- We enhanced credibility through member checking, asking participants to check interview summaries and confirm that our interpretations aligned with their lived experiences
		To look for consistent trends across various data streams	- - To improve the dependability of our analysis, we used consensus coding. This process confirmed that all researchers applied the codebook consistently, minimizing individual bias
			- -
Confirmability	To ensure the findings for itself without the researchers' influence, making the findings easier to confirm	To take aa step back to critique how the research was conducted	- - We strengthened the study's dependability and credibility through individual journaling and weekly team meetings, ensuring a consistent, critically reflexive approach
			- -
Transferability	To enable the application of these findings to different settings or populations	Sampling until theoretical saturation is reached	- Data collection ended when the team agreed that informational redundancy was achieved, meaning new interviews were no longer yielding novel codes or further developing existing themes as data saturation<br>- Participants' detailed accounts facilitated a compact description, supporting our analysis in the contextual interpretations defining their experiences as breast cancer women
